# Propagation characteristics of P-wave incident on a single uncoupled joint based on g-λ model

**DOI:** 10.1371/journal.pone.0311359

**Published:** 2024-12-05

**Authors:** Zhanfeng Fan, Qiang Xie

**Affiliations:** 1 School of Architecture and Civil Engineering, Chengdu University, Chengdu, China; 2 Sichuan Engineering Research Center for Mechanical Properties and Engineering Technology of Unsaturated Soils, Chengdu University, Chengdu, China; 3 School of Civil Engineering, Southwest Jiaotong University, Chengdu, China; Khalifa University of Science and Technology, UNITED ARAB EMIRATES

## Abstract

This paper theoretically explores the propagation attenuation of normally incident P-waves on a single uncoupled joint exhibiting nonlinear deformation behavior. The stress-deformation model of the single uncoupled joint (g-λ model with λ ≥ 1) is employed to depict the nonlinearity of uncoupled joints, with a greater value of the parameter λ signifying a lower degree of non-linearity in the joint model curve. By making use of the characteristic line approach in conjunction with the discontinuous displacement model, we have obtained the finite difference expressions which precisely represent the particle velocity and energy transmission coefficient of the transmitted wave. The expressions for the stiffness transmission coefficient and the stiffness reflection coefficient, which can effectively reflect the nonlinear variation in joint stiffness, have been accomplished. Parametric investigations are carried out to examine the impacts of nonlinear joint normal deformation on P-wave transmission. The findings suggest that when λ is respectively equal to 4.19, 8.57, 10, and 12.15, the peak particle velocity (PPV) of the transmitted waves is significantly close to the incident wave amplitude. Furthermore, when λ is fixed, the energy transmission coefficient increases with the incident wave amplitude but decreases with the incident wave frequency. The stiffness transmission coefficient rises while the stiffness reflection coefficient drops with the increasing joint closure. And if the value of λ assumes larger values, the distortion in the shape of the transmitted wave is associated with the plastic deformation in the uncoupled rock mass. These results broaden the application scope of the g-λ model and can be employed to identify fault positions in jointed rock masses during the advanced geological prediction of mountain tunnels.

## 1 Introduction

The rock mass within the Earth is filled with a large number of discontinuities, such as joints, fissures, bedding planes, faults, pores, and cavities, and these all exert a remarkable influence on its mechanical behavior [1, 2]. Comprehending the stress wave propagation within either coupled or uncoupled jointed rock masses is extremely crucial for their dynamic stability [3–5]. In spite of the previous investigations carried out on the wave propagation from intact rock masses to jointed rock masses, the stress wave attenuation resulting from reflection and transmission on jointed rock masses remains a matter that still requires to be resolved [6–11]. This deficiency in understanding has veiled the law that governs the transmission, reflection, scattering, and stress wave attenuation in jointed rock masses [12, 13]. Consequently, this paper will focus on the propagation characteristics of the P-wave incident on a single uncoupled jointed rock mass.

**Fig 1:**
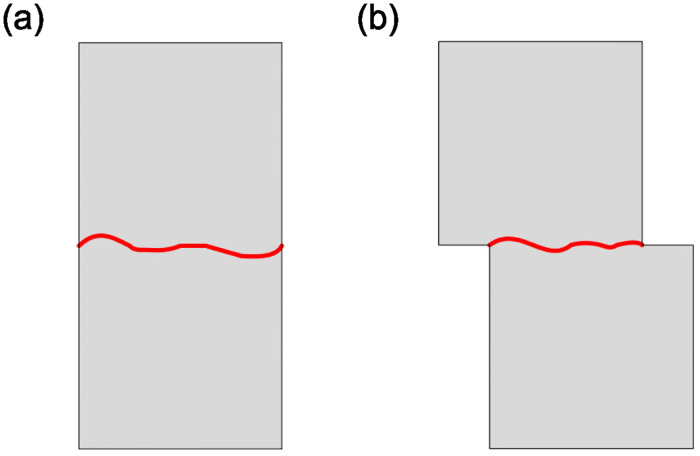
Rock with coupled and uncoupled joints. (a) A single coupled joint (b) A single uncoupled joint.

The stress-deformation models of the joints, both coupled and uncoupled, are illustrated in Fig 1. The coupled or interlocked joint refers to the complete contact between two rocks on the joint surfaces, while the uncoupled or mismatched joint indicates the presence of dislocations in the intact rocks on both sides of the joint surface [14–16]. For the coupled joint, it is recommended to utilize a hyperbolic constitutive model termed the Barton-Bandis model, also referred to as the BB Model, for depicting joint deformation. This model represents the normal deformation under the normal stress using initial stiffness along with considering size effect and maximum deformation of joints. As for the uncouple joints, a logarithm formula may provide the best-fitting relationship [17]. Currently, there are two primary methods for studying joint closure properties. The first method involves conducting indoor experiments by performing static or quasi-static loading tests on natural or artificial jointed rock masses to obtain a constitutive model of joint deformation, such as the BB model. The second method utilizes mathematical functions to fit the joint closure curve, also known as the empirical formula method, and then determines parameter values based on constraint conditions and assigns specific physical meanings such as the incremental constitutive model of discontinuities [18], the generalized semi-empirical exponential model [19], the 3-parameter constitutive model [20], the thin-layer interface model for filling and rough-surfaced joints [21], and the g-λ model of dry and unfilled joints (0 < λ < + ∞) [17]. The g-λ model is not only suitable for coupled joints (0 < λ < 1), but also applicable to uncoupled joints (λ ≥ 1). Although the parameters in the empirical formula method may be difficult to explain, they are easy to obtain.

The dynamic mechanical properties are subject to complex experimental conditions, and their research still needs to be systematic and comprehensive. The static BB model has been modified into a dynamic BB model and applied to one-dimensional stress wave propagation when the deformation of joints is not sensitive to the loading rate [7, 22]. Following this perspective, a great number of scholars have investigated the impact of joints on the stress wave propagation by employing various joint constitutive models. There exist three situations when stress waves propagate across joints. The first situation is that both the stress and the displacement on the joint surface are continuous, which is referred to as the linear displacement continuity model. The second case is that the stress is continuous while the displacement is discontinuous, known as the displacement discontinuity model (DDM) [23, 24]. This model is applicable to dry, smooth, and unfilled joints. The third scenario is that both the displacement and the stress are discontinuous, which is relevant to wet and filled joints. Utilizing the DDM, extensive research has been conducted on the nonlinear behavior of P-waves passing through either a single coupled joint or parallel multiple joints [7, 25, 26]. Stress wave traveling across a filled joint with different loading/unloading behavior was presented by introducing two BB models [27]. Under loading and unloading circumstances, the BB model was utilized to depict the propagation traits of stress waves in filled joints [12, 13]. The opening and closing characteristics of joints as well as the interaction between elastic waves have also been investigated [28]. It has been verified that the g-λ model possesses broader applicability in wave propagation compared to the BB model [29]. The modified g-λ model has been employed to analyze the propagation and attenuation of P-waves while taking into account the in situ stress in jointed rock masses [11]. However, there have been no reports on utilizing the g-λ model (λ ≥ 1) for uncoupled joints in terms of wave propagation.

A considerable quantity of indoor experiments was carried out by utilizing the Split Hopkinson Pressure Bar (SHPB) for the purpose of researching wave propagation in joints with diverse properties. For instance, the impact of the joint contact area and the spatial geometry of the joint surfaces of the dynamic characteristics as well as the wave propagation of rock joints was investigated using SHPB [30]. A novel way was to substitute the SHPB incident and output rods with gypsum and analyze the impacts of joint roughness, joint stiffness, and other factors on the propagation energy of stress waves [31]. Additionally, a Split Shear Plates model was proposed to explore the effects of a filled joint on shear wave attenuation [32]. Furthermore, the viability and effectiveness of utilizing prismatic rock samples in the SHPB experiments were also examined [33]. The effect of the number of coupled joints on the dynamic compressive strength, fragmentation effect, stress wave propagation, and energy evolution of rock masses has been researched through SHPB and LS-DYNA [34]. In recent years, several scholars have developed a bidirectional Hopkinson pressure bar (BHPB) system, designed to investigate the dynamic properties and failure characteristics of stress wave propagation in jointed rock masses under the influence of confining pressure [35]. The impacts of multiple reflections within the joints on wave propagation across layered rock masses were addressed by the governing equations for time domain-based wave propagation using an equivalent layer model [36]. Whether in terms of experiments or numerical simulation calculations, these studies focus more on the analysis of coupled joint characteristics, while the study of uncoupled joints is greatly overlooked.

In this paper, we first introduce the g-λ model (λ ≥ 1) for a single coupled joint and outline its applicable conditions. Subsequently, we derive a finite difference formula suitable for the numerical calculation of the transmitted wave particle velocity based on the characteristic line approach and the DDM. The definitions of the stiffness transmission coefficient and the stiffness reflection coefficient are initially put forward. Finally, parametric studies are carried out to obtain a deeper understanding of the effects of the g-λ model on P-wave transmission, considering joint stiffness, incident wave amplitude, frequency, and the like.

## 2 Theoretical formulations

### 2.1 g-λ model for a single uncoupled joint

The g-λ model (0 < λ < + ∞) serves as a regular manifestation of the deformation of a joint when subjected to normal effective stress, and it has the capability to be implemented in both coupled and uncoupled joints [17]. The g-λ model can be represented by the following equation,

$$\begin{array}{c} d_{n}=d_{ma}[1-{\left(\frac{\lambda \sigma_{n}}{d_{ma}k_{ni}}+1\right)}^{-\frac{1}{\lambda }}] \end{array}$$ (1)

where *d*_*n*_ and *d*_*ma*_ respectively signify the joint closure and the maximum allowable closure of the joint. Meanwhile, *k*_*ni*_ represents the initial joint stiffness under the normal effective stress, and *σ*_*n*_ stands for the normal effective stress. One of the prominent characteristics of the g-λ model lies in the incorporation of a parameter λ (in a dimensionless unit), which is employed to expedite the pace of normal deformation by building upon the BB model and the traditional exponential model.

In the g-λ model, there exist three parameters, namely *d*_*ma*_, *k*_*ni*_, and λ. The values of *d*_*ma*_ and *k*_*ni*_ can be ascertained through laboratory measurements [15, 16]. The parameter λ is related to the degree of weathering, roughness, undulation, matching of the joint surfaces, and the strength of the rock joint walls. Specifically, the g-λ model is structured in such a way that it takes into account various factors and relationships to accurately represent the deformation process. It carefully considers how the joint behaves under normal effective stress and how the addition of the parameter λ influences and enhances the rate of deformation. This model provides a more comprehensive and detailed understanding of the joint deformation characteristics, allowing for more precise analysis and prediction in relevant applications.

**Fig 2:**
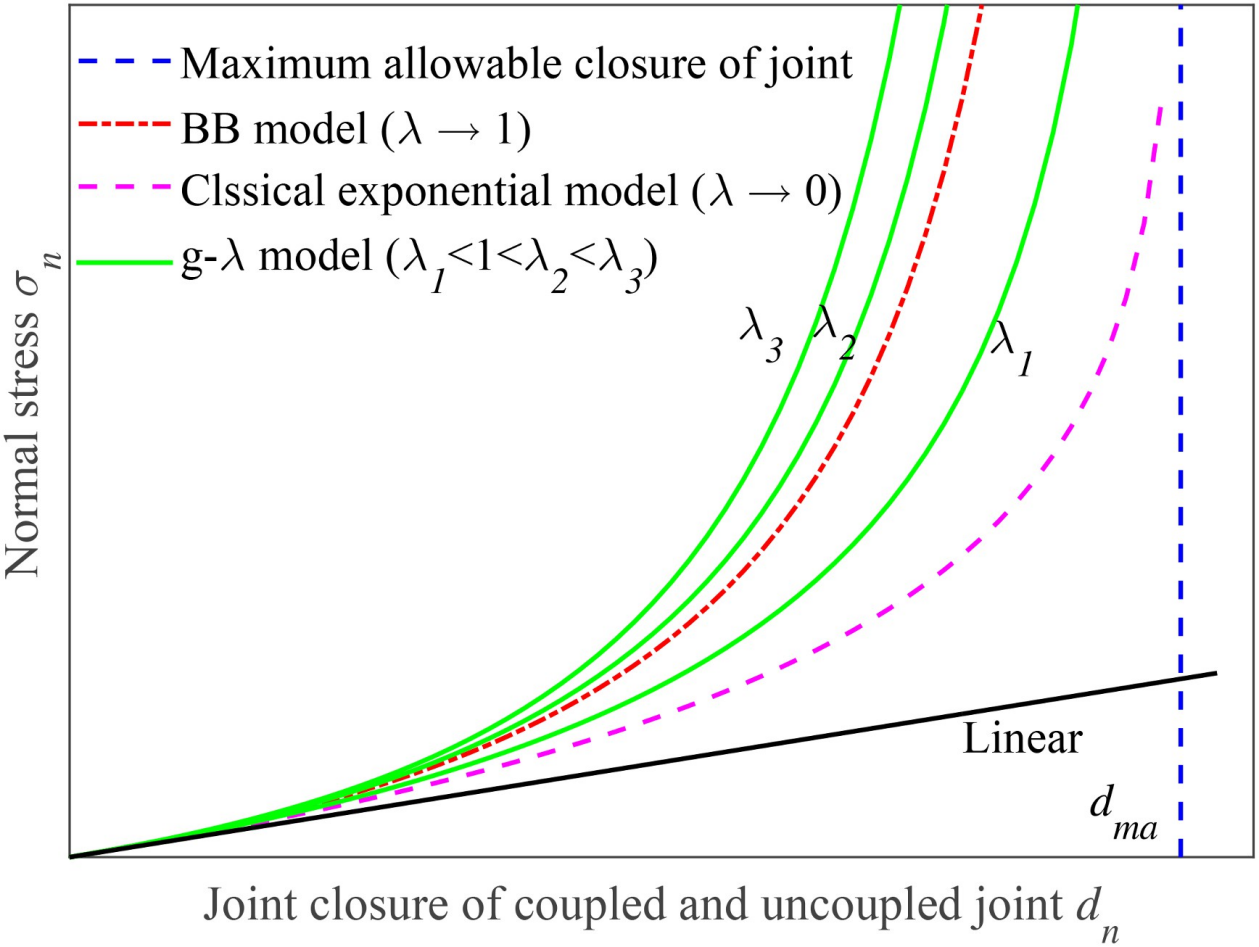
Scheme of g-λ model with different parameter λ (0 < λ < + ∞).

It is noted that the larger the value of λ, the lower the degree of non-linearity exhibited by the joint model curve, as shown in Fig 2. When λ is relatively small, the joint model curve may show more significant non-linear behavior, with more pronounced deviations from a linear trend. This relationship between λ and the non-linearity of the joint model curve is crucial for understanding and analyzing the behavior of the joint. In the present study, we only consider the case of λ ≥ 1 for a single uncoupled joint because the g-λ model (0 < λ < 1) for the coupled joints has been discussed in Refs. [11, 29].

In the case where λ = 1, the g-λ model undergoes degeneration and transforms into the BB model as follows,

$$\begin{array}{c} d_{n}=\frac{\sigma_{n}}{k_{ni}+\sigma_{n}/d_{ma}} \end{array}$$ (2)

In accordance with L’Hospital’s rule, when λ approaches positive infinity, the exponential term in Eq (1) can be obtained in the following way,

$$\begin{array}{cc} \lim_{\lambda \to +\infty }{\left(\frac{\lambda \sigma_{n}}{k_{ni}d_{ma}}+1\right)}^{-\frac{1}{\lambda }} & =\text{exp}\;\underset{\lambda \to +\infty }{\{\text{lim}\;}[-\frac{1}{\lambda }\text{ln}\;(\frac{\lambda \sigma_{n}}{k_{ni}d_{ma}}+1)]\}\\ & =\text{exp}\;(-\frac{\sigma_{n}}{\lambda \sigma_{n}+k_{ni}d_{ma}}) \end{array}$$ (3)

Substituting Eq (3) into Eq (1), we can derive

$$\begin{array}{c} d_{n}{|}_{\lambda \to +\infty }=d_{ma}[1-\text{exp}\;(-\frac{\sigma_{n}}{\lambda \sigma_{n}+k_{ni}d_{ma}})]=0 \end{array}$$ (4)

From a mathematical point of view, the derivation of Eq (4) is accurate. Nevertheless, the physical significance of Eq (4) still requires further elaboration, and we will discuss the value of λ in subsequent sections.

**Fig 3:**
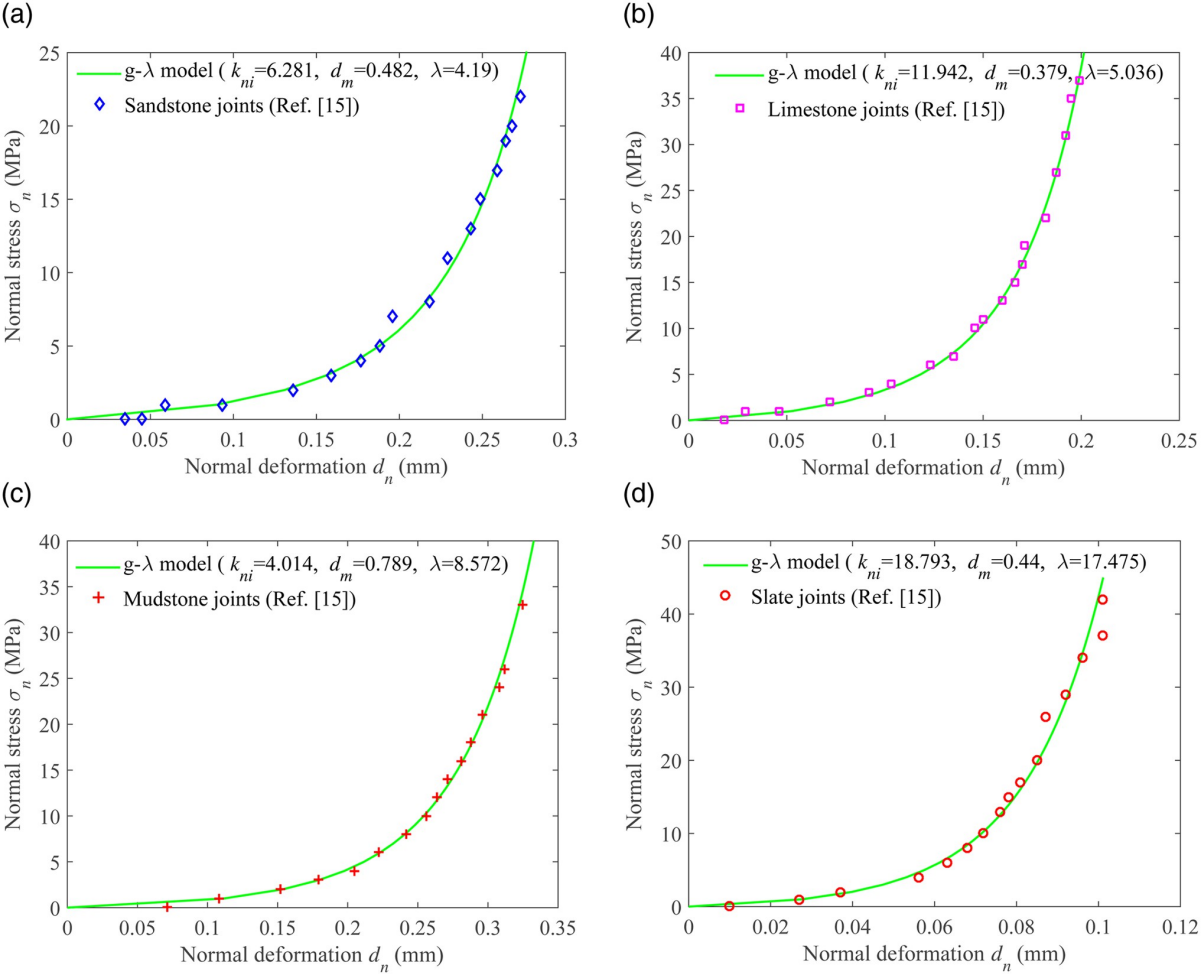
Fitting the experimental data of uncoupled joint closure of four different types of rock mass joints under normal stress using the g-λ model. (a) Sandstone joints (b) Limestone joints (c) Mudstone joints (d) Slate joints.

In order to verify that the g-λ model can better describe the closure properties of uncoupled joints, we compared the fitting results of the g-λ model with the experimental results of four existing types of rock mass joints. These four types of rock joints are sandstone joints, slate joints, limestone joints, and mudstone joints [15]. The fitting results are shown in Fig 3. It can be seen that the g-λ model can fit the experimental data well, with values of λ being 4.19, 5.036, 8.572, and 17.475, respectively.

### 2.2 The method of characteristics

It is hypothesized that a joint is present at the location *x* = *x*_1_ within a half-space that is characterized by linear elasticity, homogeneity, and isotropy. When a plane P-wave with normal incidence strikes the joint, both the reflected wave and the transmitted wave will be produced. According to DDM, the stress and displacement on both sides of the joint can be expressed as follows,

$$\begin{array}{c} \left\{ \begin{array}{c} \sigma \left(x_{1},t\right)=\sigma \left(x_{1}+d_{n},t\right)\\ u\left(x_{1},t\right)-u\left(x_{1}+d_{n},t\right)=d_{ma}[1-{\left(\frac{\lambda \sigma_{n}}{d_{ma}k_{ni}}+1\right)}^{-\frac{1}{\lambda }}] \end{array}\right. \end{array}$$ (5)

where *σ*(*x*_1_, *t*) and *σ*(*x*_1_ + *d*_*n*_, *t*) respectively represent the normal stress before and after the P-wave passes through the joint. *u*(*x*_1_, *t*) and *u*(*x*_1_ + *d*_*n*_, *t*) respectively signify the displacement before and after the P-wave traverses the joint. Specifically, the continuous nature of stresses implies that there is a seamless transition of stress values across the joint. This indicates that the stresses acting on one side of the joint are smoothly carried over to the other side without any abrupt changes or discontinuities. On the other hand, the equality of the difference in displacements in the closure or opening of the joint reflects the fact that the joint experiences a certain amount of deformation or movement.

By taking the derivative of the displacement in Eq (5) with respect to time, it can be inferred that the particle velocity before and after the stress wave passes through the single uncoupled joint is presented as follows,

$$\begin{array}{cc} \frac{\partial u(x_{1},t)}{\partial t}-\frac{\partial u(x_{1}+d_{n},t)}{\partial t} & =v\left(x_{1},t\right)-v\left(x_{1}+d_{n},t\right)\\ & =\frac{{\left(\frac{\lambda \sigma_{n}}{d_{ma}k_{ni}}+1\right)}^{-\frac{\lambda +1}{\lambda }}}{k_{ni}}\frac{\partial \sigma_{n}}{\partial t} \end{array}$$ (6)

**Fig 4:**
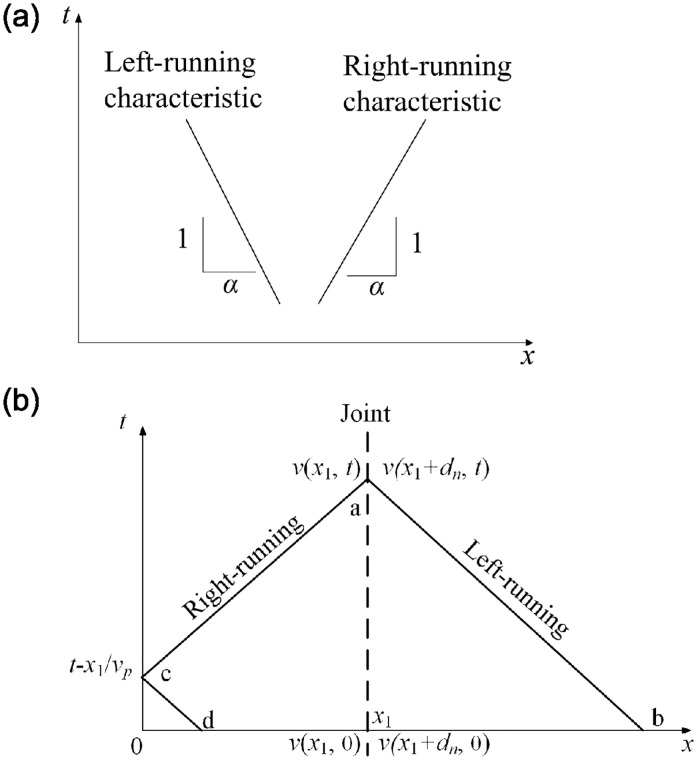
Illustration of the method of characteristics for solving the one-dimensional wave equation in the *x*-*t* plane [7, 37, 38]. (a) Right- and left-running characteristics in the *x*-*t* plane. (b) The corresponding features of a one-dimensional wave incident on the joint in a half-space on the *x*-*t* plane.

The Method of Characteristics (MC) has found extensive application in resolving the issues related to one-dimensional wave propagation within linearly elastic joint, as shown in Fig 4. The straight lines having a slope of *α* and -*α* in the *x*-*t* plane are referred to as the right-running and left-running characteristics of the one-dimensional wave equation, as shown in Fig 4(a). Along right-running line with slope *α* in the *x*-*t* plane, the following expression holds

$$\begin{array}{c} zv(x,t)+\sigma (x,t)=\text{constant} \end{array}$$ (7)

Similarly, along left-running line with slope -*α* in the *x*-*t* plane, the following expression satisfies

$$\begin{array}{c} zv(x,t)-\sigma (x,t)=\text{constant} \end{array}$$ (8)

where *z* is the acoustic impedance of the rock, *v*(*x*, *t*) is the particle velocity and *σ*(*x*, *t*) is the dynamic stress.

According to Fig 4(a), it can be seen that lines ab and cd in Fig 4(b) are the left -running lines, while line ac is the right-running line. Therefore, along left-running characteristic line ab,

$$\begin{array}{c} zv\left(x_{1}+d_{n},t\right)-\sigma \left(x_{1},t\right)=0 \end{array}$$ (9)

Similarly, along the right-running characteristic line ac,

$$\begin{array}{c} zv\left(x_{1},t\right)+\sigma \left(x_{1},t\right)=zp\left(t-x_{1}/v_{p}\right)+\sigma \left(0,t-x_{1}/v_{p}\right) \end{array}$$ (10)

where *p*(*t* − *x*_1_/*v*_*p*_) is the particle velocity input to the boundary at time *t* − *x*_1_/*v*_*p*_, and *σ*(0, *t* − *x*_1_/*v*_*p*_) is the stress for *x*_1_=0 at time *t* − *x*_1_/*v*_*p*_.

Along another left-running characteristic line cd,

$$\begin{array}{c} zp\left(t-x_{1}/v_{p}\right)-\sigma \left(0,t-x_{1}/v_{p}\right)=0 \end{array}$$ (11)

Summing up Eqs (10) and (11) yields

$$\begin{array}{c} zv\left(x_{1},t\right)+\sigma \left(x_{1},t\right)=2zp\left(t-x_{1}/v_{p}\right) \end{array}$$ (12)

When considering the DDM and the MC, $\frac{\partial v(x_{1}+d_{n},t)}{\partial t}$ can be deduced as follows,

$$\begin{array}{cc} \frac{\partial v(x_{1}+d_{n},t)}{\partial t}= & \frac{2k_{ni}}{z}[p\left(t-x_{1}/v_{p}\right)-v\left(x_{1}+d_{n},t\right)]\cdot \\ & \;{\left[\frac{\lambda zv(x_{1}+d_{n},t)}{d_{ma}k_{ni}}+1\right]}^{\frac{\lambda +1}{\lambda }} \end{array}$$ (13)

where *p*(*t* − *x*_1_/*v*_*p*_) is the particle velocity that was inputted to the boundary at the time *t* − *x*_1_/*v*_*p*_.

Based on Eqs (9) and (12), the relation between the particle velocity before and after the joint can be expressed as,

$$\begin{array}{c} v\left(x_{1},t\right)+v\left(x_{1}+d_{n},t\right)=2p\left(t-x_{1}/v_{p}\right) \end{array}$$ (14)

Substituting Eqs (6) and (9) into Eq (14), we can derive $\frac{\partial v(x_{1}+d_{n},t)}{\partial t}$ as follows,

$$\begin{array}{cc} \frac{\partial v(x_{1}+d_{n},t)}{\partial t}= & \frac{2k_{ni}}{z}[p\left(t-x_{1}/v_{p}\right)-v\left(x_{1}+d_{n},t\right)]\cdot \\ & \;{\left[\frac{\lambda zv(x_{1}+d_{n},t)}{d_{ma}k_{ni}}+1\right]}^{\frac{\lambda +1}{\lambda }} \end{array}$$ (15)

The detailed derivation process of Eq (15) can be found in the S1 Appendix.

For the purpose of computation calculation, Eq (13) is presented in the finite difference form as follows,

$$\begin{array}{ll} v\left(x_{1}+d_{n},t_{j+1}\right) & =v\left(x_{1}+d_{n},t_{j}\right)+\\ \; & \frac{2k_{ni}}{z}\left[p\left(0,t-x_{1}/v_{p}\right)-v\left(x_{1}+d_{n},t_{j}\right)\right].\\ \; & {\left[\frac{\lambda zv\left(x_{1}+d_{n},t_{j}\right)}{d_{ma}k_{ni}}+1\right]}^{\frac{\lambda +1}{\lambda }}\Delta t \end{array}$$ (16)

where Δ*t* is the time interval.

Furthermore, the energy of transmitted waves is inspected through the energy transmission coefficient *T*_*e*_, which is defined as follows,

$$\begin{array}{c} T_{e}=\frac{E_{tra}}{E_{inc}}=\frac{\sum_{j=t_{tra}^{0}}^{j=t_{tra}^{0}+T_{tra}}z{\left(v_{tra}\left(x_{1},t_{j}\right)\right)}^{2}\Delta t}{\sum_{j=t_{inc}^{0}}^{j=t_{inc}^{0}+T_{inc}}z{\left(v_{inc}\left(x_{1},t_{j}\right)\right)}^{2}\Delta t} \end{array}$$ (17)

where *E*, *T*, and *t* are the energy, period, and initial time of the stress wave, respectively. The subscripts ‘*tra*’ and ‘*inc*’ represent transmission and incidence, respectively, while the superscript ‘0’ represents the initial time. For example, $t_{tra}^{0}$ and $t_{inc}^{0}$ respectively are the initial times of the transmitted and incident waves [7, 26, 27].

### 2.3 Stiffness transmission coefficient and stiffness reflection coefficient

Transmission coefficient *T*_*lin*_ and reflection coefficient *R*_*lin*_ for a wave incident perpendicularly to a linear deformation joint in the same rock can be computed as follows [23, 24]

$$\begin{array}{c} T_{lin}={\left[\frac{4{\left(\frac{k_{ni}}{z\omega }\right)}^{2}}{4{\left(\frac{k_{ni}}{z\omega }\right)}^{2}+1}\right]}^{1/2} \end{array}$$ (18)

$$\begin{array}{c} R_{lin}={\left[\frac{1}{4{\left(\frac{k_{ni}}{z\omega }\right)}^{2}+1}\right]}^{1/2} \end{array}$$ (19)

where *ω* = 2*πf* is the angular wave frequency and *f* is the frequency of the incident wave.

In the g-λ model, the flexibility of the joint *C*_*n*_ is defined as follows,

$$\begin{array}{c} C_{n}=\frac{1}{K_{n}}=\frac{\partial d_{n}}{\partial \sigma_{n}}=\frac{1}{k_{ni}}{\left(1-\frac{d_{n}}{d_{ma}}\right)}^{\lambda +1} \end{array}$$ (20)

where *K*_*n*_ is the equivalent stiffness as mentioned in Ref. [17]. In an effort to acquire the transmission coefficient and reflection coefficient for the g-λ model, we make use of the fundamental concept of the Lemaitre equivalent strain assumption within damage mechanics as described in Ref. [39]. And we assume that ${\left(1-\frac{d_{n}}{d_{ma}}\right)}^{\lambda +1}$ serves to represent the nonlinear coefficient of the joint stiffness. The variable *k* in Eqs (18) and (19) can be substituted by the *K*_*n*_ in Eq (20). Therefore, the expressions of the stiffness transmission coefficient *T*_*stc*_ and the stiffness reflection coefficient *R*_*src*_ can be expressed as follows,

$$\begin{array}{c} T_{stc}=\frac{1}{\sqrt{{\left[\frac{z\omega {\left(1-\gamma \right)}^{\lambda +1}}{2k_{ni}}\right]}^{2}+1}} \end{array}$$ (21)

$$\begin{array}{c} R_{src}=\frac{1}{\sqrt{{\left[\frac{2k_{ni}}{z\omega {\left(1-\gamma \right)}^{\lambda +1}}\right]}^{2}+1}} \end{array}$$ (22)

where $\gamma =\frac{d_{n}}{d_{ma}}$ is the ratio of the joint closure to the maximum allowable joint closure. The stiffness transmission coefficient and the stiffness reflection coefficient can effectively reflect the nonlinear variation in joint stiffness. It is discovered that the *T*_*stc*_ and *R*_*src*_ are in accordance with the corresponding outcomes when *γ* is equal to 0 as cited in Refs. [23, 24].

## 3 Model comparison

It is of great importance and necessity to conduct a comparison between the g-λ model and the existing nonlinear joint closure models in order to validate the accuracy of the derived formulas presented in Section 2. The BB model and a 3-parameter constitutive model were deliberately chosen as the objects for comparison. The BB model is a renowned and classical hyperbolic model, which is clearly demonstrated in Eq (2). Meanwhile, the 3-parameter constitutive model was put forward with the intention of enhancing and addressing the mathematical flaws that exist in both the BB model and the classical exponential model. This is because both of these models tend to show significant deviations from the experimental results when it comes to the median stress level, as mentioned in Ref. [20]. And its specific expression is presented as follows,

$$\begin{array}{c} d_{n}=\xi d_{ma}\{\text{exp}\;[\frac{\left(1-\xi \right)\sigma_{n}}{\xi k_{ni}d_{ma}}]-1\}/\{\text{exp}\;[\frac{\left(1-\xi \right)\sigma_{n}}{\xi k_{ni}d_{ma}}]-\xi \} \end{array}$$ (23)

where *ξ* is the correction coefficient for the joint closure and *ξ* ∈ (1, + ∞). Other symbols have the same meaning as the Eq (1).

Without loss of generality, the half-sine P-wave is selected to be the incident wave. The frequency is 50 Hz. The reason for choosing this specific P-wave is that it will not bring about any damage to the jointed rock mass. During the numerical simulation procedure, the key point lies in analyzing the propagation characteristics of those wave amplitude thresholds, for instance, the peak particle velocity (PPV). The incident P-wave is presented in the following way,

$$\begin{array}{cc} & v_{inc}\left(0,t\right)=\{\begin{array}{cc} A_{0}\sin\left(2\pi ft\right)\;\; & \text{for}\;0\leq t\leq \frac{1}{2f}\\ 0\;\; & \text{otherwise,} \end{array} \end{array}$$ (24)

where *v*_*inc*_(0, *t*) represents the incident wave velocity at the time *t* and the position *x*_1_ being equal to 0. *A*_0_ is the incident wave amplitude. It is worthy of being noted that the comparison of the PPV utilizes the parameters of coupled joints, since both the BB model and the 3-parameter constitutive model describe the characteristics of the normal deformation of coupled joints. The parameters of the coupled jointed rock mass are presented in Table 1.

**Table 1:** Key parameters of a coupled jointed rock mass [7].

Parameter Description	Symbol	Value	Unit
Density of rock	*ρ*	2400	kg/m^3^
Wave velocity of rock	*v*_*p*_	4500	m/s
Initial normal stiffness of joint	*k*_*ni*_	1.25	GPa/m
Maximum allowable	*d*_*ma*_	0.61	mm
closure of joint			

**Fig 5:**
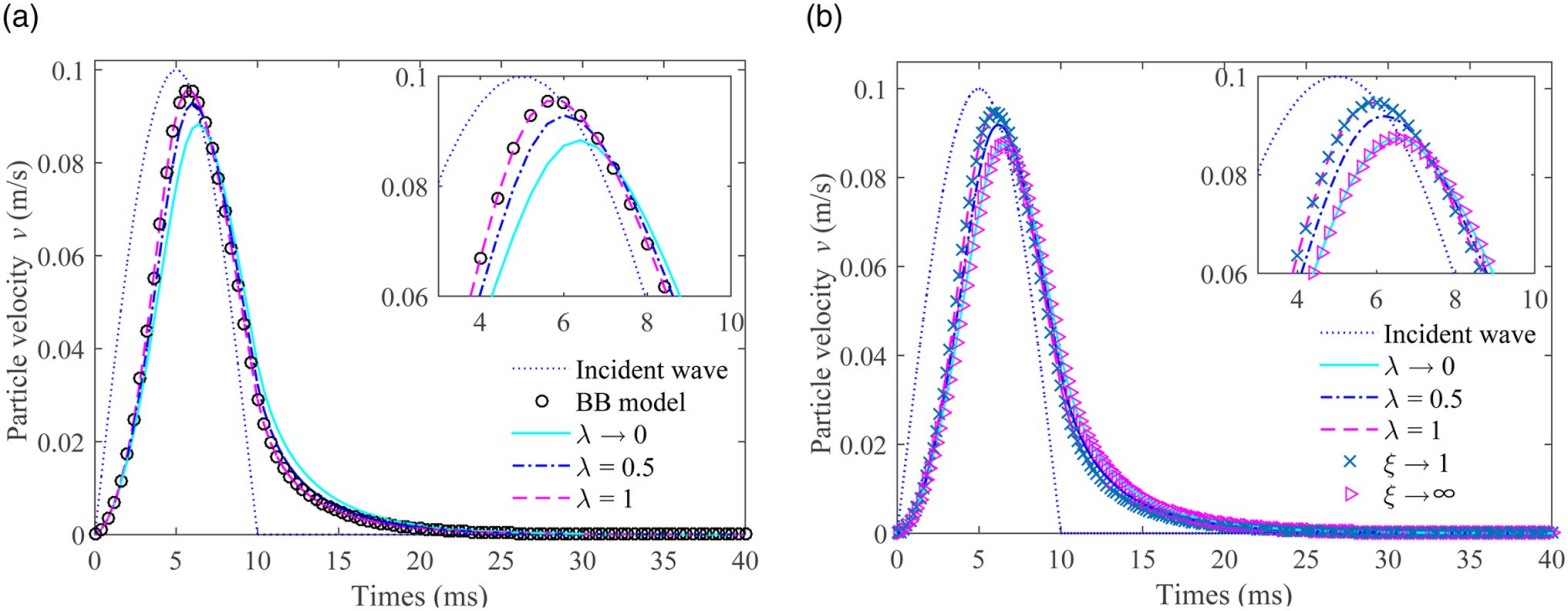
The particle velocities of the transmitted wave based on the g-λ model, the BB model, and the 3-parameter constitutive model. (a) Compared with BB model (b) Compared with 3-parameter model.

The particle velocities of transmitted waves were compared based on the g-λ model, BB model, and 3-parameter constitutive model, as shown in Fig 5. It can be observed that the shape of the transmitted wave bears a resemblance to that of the incident wave, though with a certain phase delay. The PPV gradually rises as the value of λ goes from 0 to 1. When λ approaches 1, the PPV of the transmitted waves for both the g-λ model and the BB model is precisely the same, as shown in Fig 5(a). In Fig 5(b), when λ is equal to 1 and *ξ* is close to 1, the PPVs of the g-λ model and the 3-parameter constitutive model are identical. A similar situation can be seen when λ is close to 0 and *ξ* is close to +∞. The comparison outcomes firmly affirm that the derived formula is accurate.

## 4 Parametric studies

In order to clearly illustrate and prove that the g-λ model can indeed be effectively applied in the context of wave propagation, we primarily focus on conducting a detailed analysis of the transmission behaviors of a normal P-wave as it traverses across a single uncoupled joint. The purpose is to gain a more comprehensive and in-depth understanding of how the g-λ model and its implications for wave propagation in the case of a single uncoupled joint. Due to the limited research data on uncoupled joints, the selection of parameters mainly comes from Refs. [15, 17]. The physical and mechanical parameters of sandstone, siltstone, slate, and limestone are shown in Table 2. The values of λ obtained by fitting the g-λ model to four types of uncoupled jointed rock masses are 4.19, 8.57, 10, and 12.15, respectively.

**Table 2:** Key parameters of uncoupled jointed rock masses [17].

	*ρ* /kg/m^3^	*v*_*p*_ /m/s	*k*_*ni*_ /GPa/m	*d*_*ma*_ /mm	λ
Sandstone	2457	3125	8.4	0.293	4.19
Siltstone	2467	3399	13.4	0.32	8.57
Limestone	2873	4130	19.1	0.193	10
Slate	2823	4835	20.3	0.159	12.15

### 4.1 Study on the particle velocity of transmitted wave

**Fig 6:**
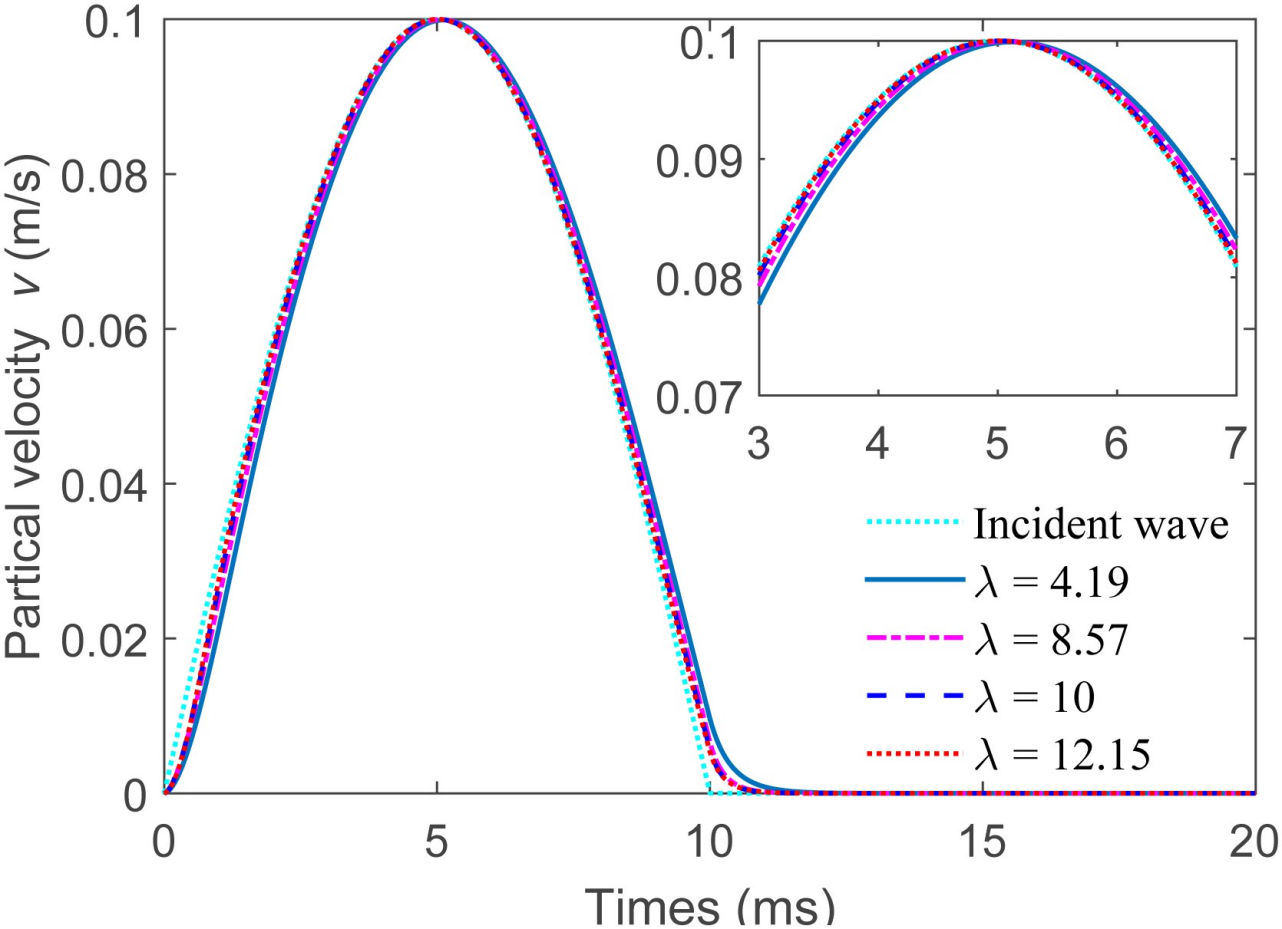
The particle velocities of transmitted waves passing through uncoupled jointed rock masses in sandstone, siltstone, limestone, and slate.

The particle velocities of the transmitted wave for sandstone, siltstone, limestone, and slate are computed in accordance with the g-λ model, as shown in Fig 6. Where *A*_0_ is equal to 0.1 m/s and *f* is equal to 50 Hz. It is noted that although the waveform of the transmitted wave bears a certain resemblance to that of the incident wave, there is a relatively slight phase delay. This particular observation is in line with the wave propagation phenomenon that occurs in coupled joints as mentioned in Ref. [29]. The PPV gradually increases along with the increase in λ (indicating lesser weathering, reduced roughness, and smaller joint closure). The PPVs of the four different rock masses, namely sandstone, siltstone, limestone, and slate, are 0.099 m/s, 0.1 m/s, 0.1 m/s, and 0.1 m/s, respectively. These findings imply that when the joint stiffness attains a certain specific level, both sides of the uncoupled joint have completely merged with enhanced coupling and transmitted waves. In other words, all the incident waves are able to pass through the uncoupled joints without encountering any reflection. Therefore, it is indeed possible and practical to make use of the g-λ model to analyze the nonlinear behavior when P-waves are propagating through single uncoupled joints.

### 4.2 Amplitude-dependence of energy transmission

**Fig 7:**
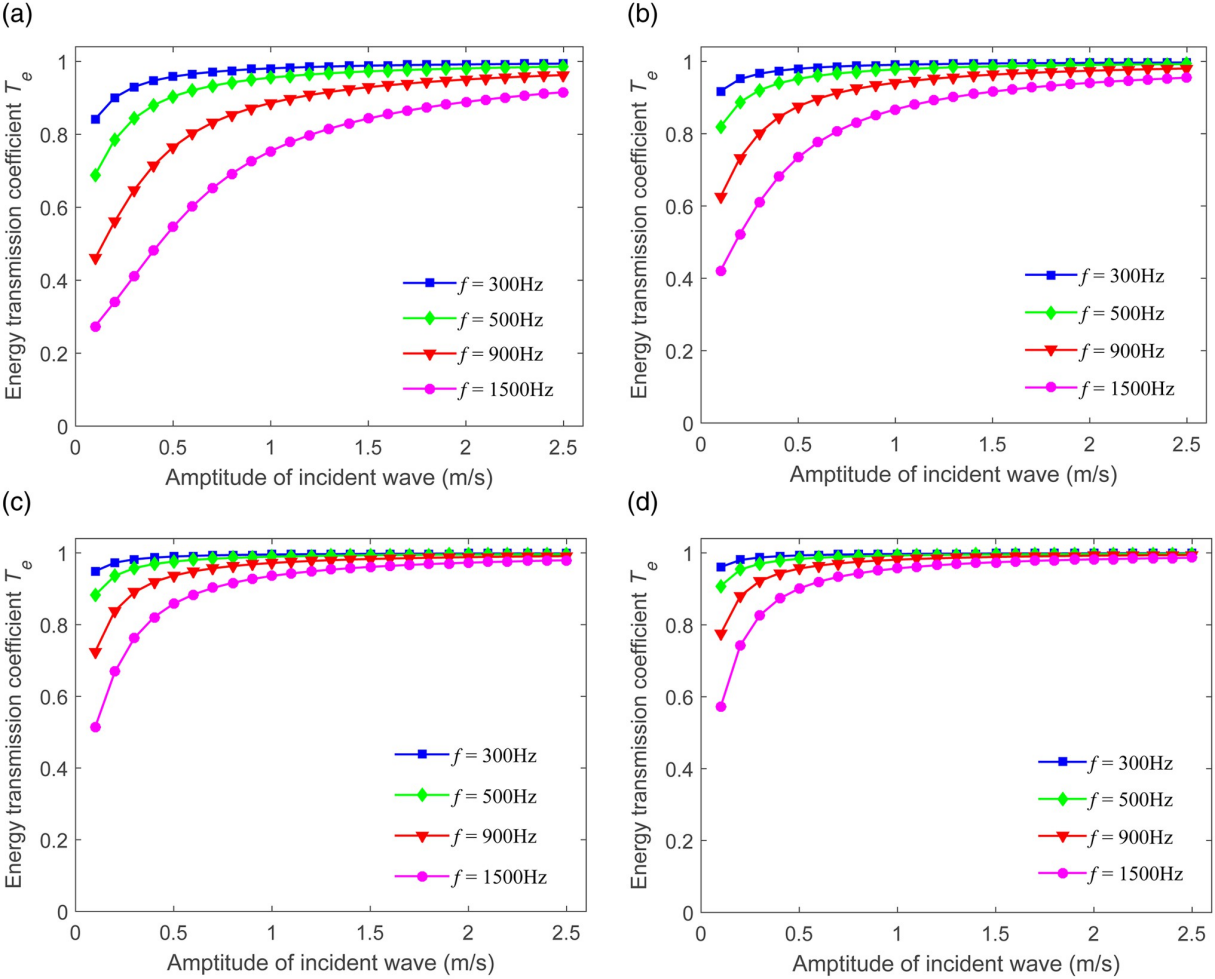
Amplitude-dependence of energy transmission for sandstone, siltstone, limestone, and slate. (a) λ=4.19 (b) λ=8.57 (c) λ=10 (d) λ=12.15.

This particular section conducts a detailed examination and exploration of the amplitude-dependence characteristics of the energy transmission *T*_*e*_. According to Eq (17), the variations of *T*_*e*_ in relation to the incident wave amplitude at different frequencies (*f*=300 Hz, *f*=500 Hz, *f*=900 Hz, and *f*=1500 Hz) are presented in Fig 7. It can be noticed that *T*_*e*_ progressively increases with the increase of the incident wave amplitude. It was also discovered that when the incident wave amplitude is fixed, *T*_*e*_ steadily decreases as the value of *f* rises from 300 Hz to 1500 Hz. These phenomena suggest that uncoupled joints also possess high-frequency filtering effects. When the value of λ escalates from 4.19 to 12.15, the energy of the transmitted wave undergoes a remarkable increase.

### 4.3 Frequency-dependence of energy transmission

**Fig 8:**
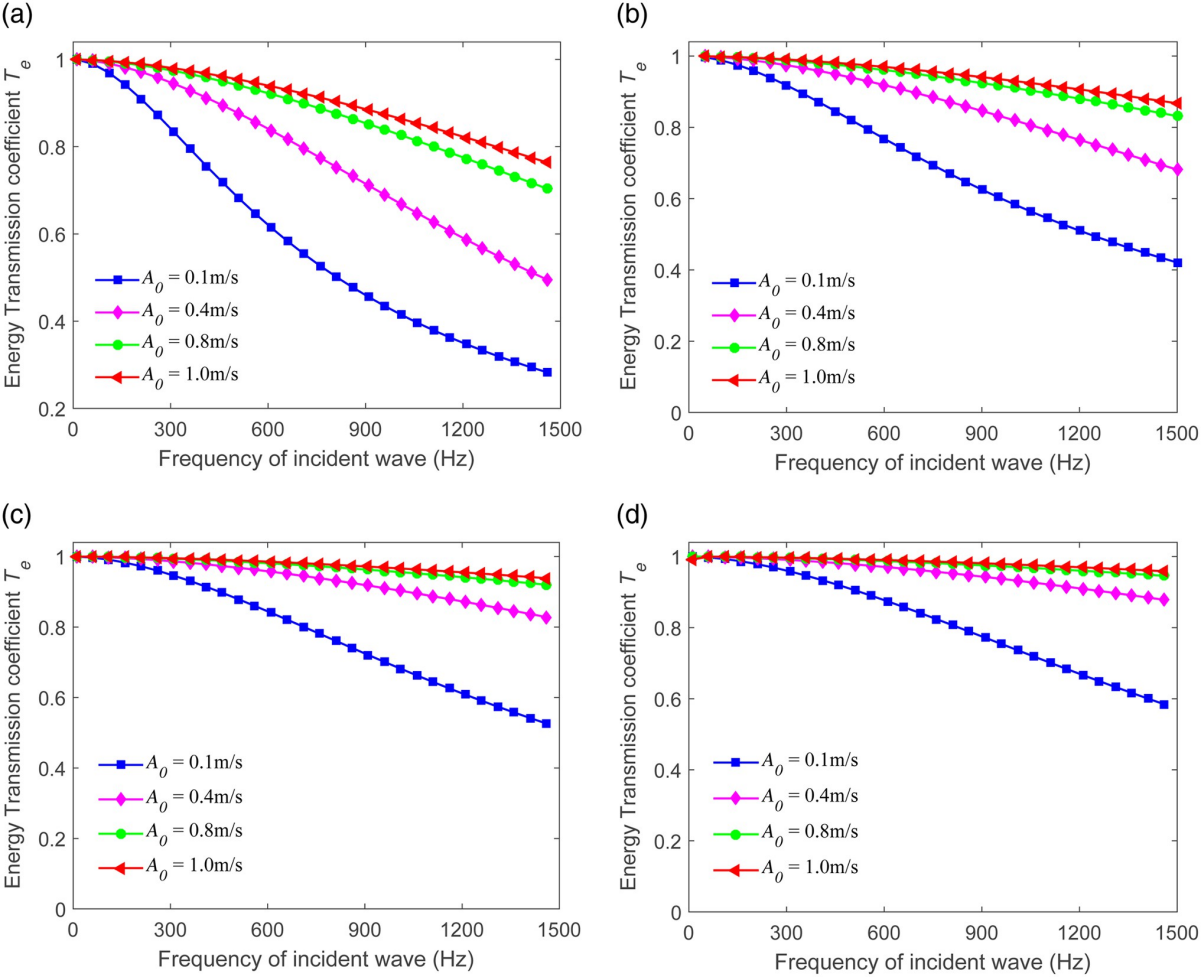
Frequency-dependence of energy transmission for sandstone, siltstone, limestone, and slate. (a) λ=4.19 (b) λ=8.57 (c) λ=10 (d) λ=12.15.

This section showcases the frequency-dependence of the energy transmission *T*_*e*_. We make the assumption that the incident waves possess diverse frequencies and four fixed amplitudes *A*_0_ (where *A*_0_ is equal to 0.1 m/s, 0.4 m/s, 0.8 m/s, and 1 m/s, respectively). The relationships between the energy transmission and the incident wave frequency are presented in Fig 8(a) to 8(d), respectively. It can be observed that *T*_*e*_ steadily decreases as the frequency escalates from 10 Hz to 1500 Hz. When the value λ is constant, *T*_*e*_ increases along with the growth of the amplitude. The transmitted energy rises as the value of λ increases from 4.19 to 12.15. This clearly indicates that an increase in the joint stiffness is conducive to enhancing the transmitted wave energy. We can see a corresponding increase in the amount of energy that is successfully transmitted through the joint considering a specific scenario where the incident wave has a certain frequency and amplitude. This implies that a more rigid joint configuration allows for a greater portion of the incident wave energy to be passed on, rather than being dissipated or reflected. Such findings have significant implications in understanding and analyzing the behavior of wave propagation in systems with varying joint stiffness and frequencies.

### 4.4 Numerical calculation of stiffness transmission coefficient and stiffness reflection coefficient

**Fig 9:**
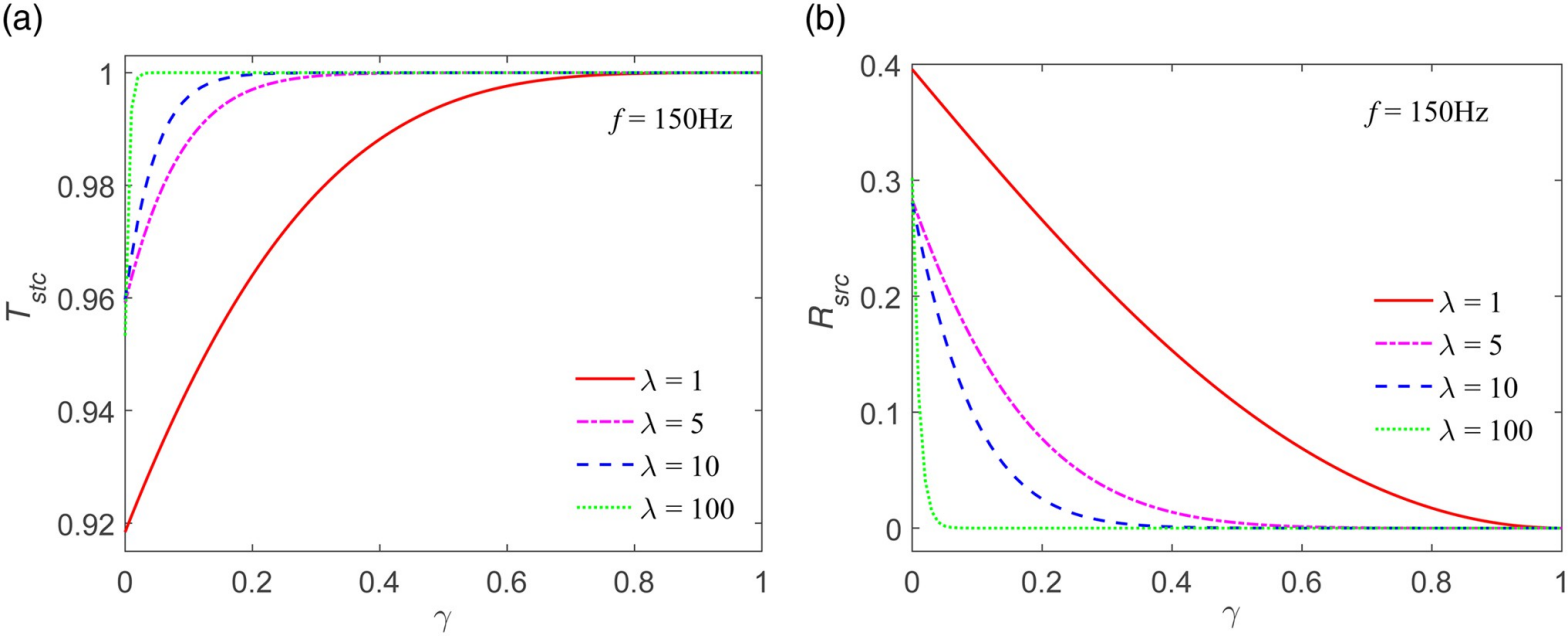
Stiffness transmission coefficient *T*_*stc*_ and stiffness reflection coefficient *R*_*src*_ as a function of *γ* for different values of λ. (a) *T*_*stc*_ vs. *γ* (b) *R*_*stc*_ vs. *γ*.

The associations between the stiffness transmission coefficient and stiffness reflection coefficient and *γ* are presented and depicted in Fig 9. The stiffness transmission coefficients *T*_*stc*_ for four distinct values of λ show an increasing trend and eventually reach 1 when *γ* rises from 0 to 1, as shown in Fig 9(a), where *f* is 150 Hz. Additionally, *T*_*stc*_ also undergoes an increase along with the growth of λ. The stiffness reflection coefficients *R*_*src*_ for four different values of λ decrease until they reach 0 as *γ* increases, as shown in Fig 9(b). It proves to be a convenient approach to deduce the transmission coefficient and the reflection coefficient. In the case where *γ* remains constant, *R*_*src*_ diminishes with the increase of λ. It is observed that the stiffness reflection coefficient at low frequencies is smaller than that at high frequencies when λ and *γ* are the same. When we analyze the specific situation where various values of λ and *γ* are considered, we can clearly see how the stiffness transmission and reflection coefficients change in response to these parameters. This understanding helps in better comprehending the behavior of wave propagation in systems with different joint characteristics.

Fig 9 reveals that as the value of λ goes up, which means the degree of joint weathering is weaker and, correspondingly, the joint stiffness is more rigid and the amount of joint closure is smaller. This situation then results in a greater quantity of transmitted waves and a lesser amount of reflected waves. What this also indicates is that the lesser the nonlinear degree of the g-λ model, the more abundant the transmission wave will be, and simultaneously, the less the reflected wave will be. For instance, when we closely examine the graphical representation in Fig 9, we can see a clear trend whereas λ steadily increases, the effects on the transmitted and reflected waves become apparent. It shows how a change in the degree of joint weathering and the associated stiffness can have a profound impact on the wave behavior.

## 5 Discussion on the range of parameter λ

**Fig 10:**
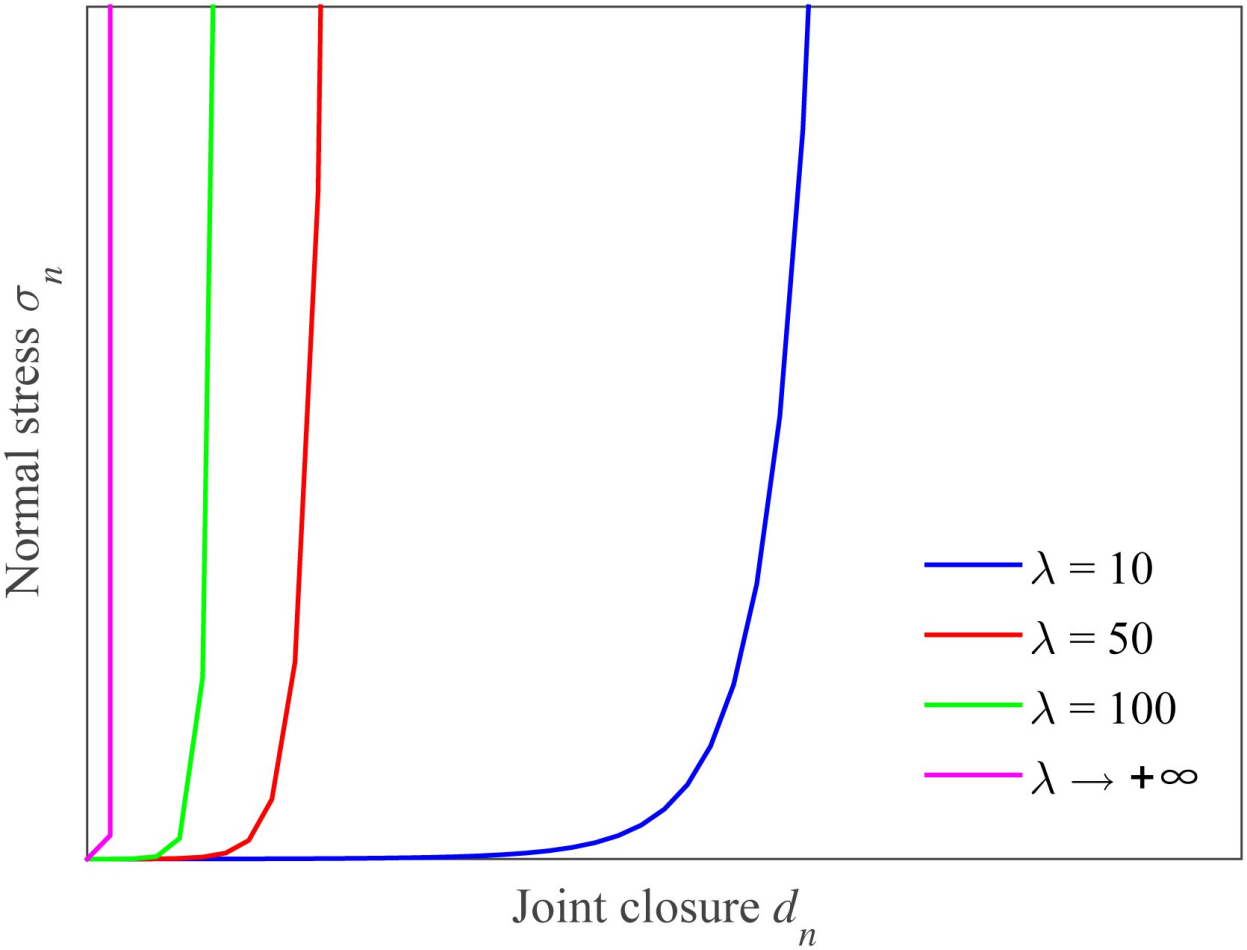
Scheme of g-λ model with larger values λ.

**Fig 11:**
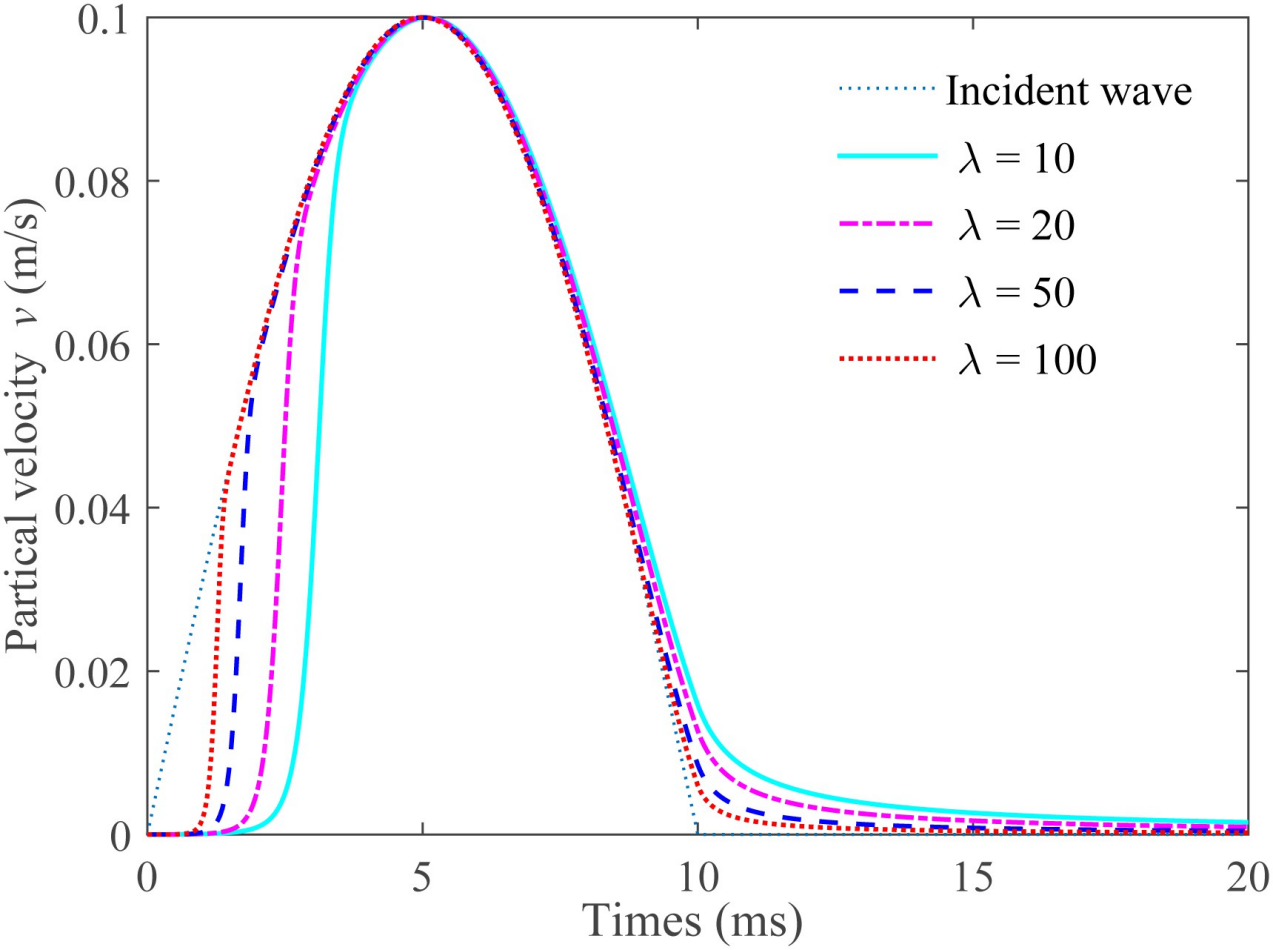
Transmitted waves with higher values of λ when the incident wave passes through a single uncoupled joint.

The previous section offers an initial verification that the g-λ model is more appropriate for the normal deformation of a single uncoupled joint when the value of λ is less than 15. As the parameter λ keeps rising, the joint stiffness will correspondingly increase, and the amount of closure will reduce. This then leads to a reduction in the degree of joint weathering, undulation, and simultaneously brings about an increase in the transmitted wave while causing a decrease in the reflected wave. Generally speaking, the parameter λ can be related to aspects such as joint weathering, roughness, the degree of fluctuation, the surface matching of joints, and the strength of the rock joint walls. When it gradually increases from 10 to an infinite value, the stress-deformation relationship follows a logarithmic variation pattern, as shown in Fig 10. For instance, taking sandstone as an example, when λ is set as 10, 20, 50, and 100, the variations in transmitted waves are presented in Fig 11. It can be observed that as λ continues to increase, there is a distortion in the transmitted wave pattern, suggesting that there is plastic deformation in the rock mass. This is because for the single uncoupled joint, there is greater plastic deformation under normal load compared to that of the single coupled joint. Hence, it can be deduced that parameter λ is associated with a relatively higher plastic work or plastic deformation of the rock joints. However, further investigations are required to firmly establish this specific relationship.

## 6 Conclusions

In this paper, the g-λ model is deliberately employed to conduct a comprehensive analysis of the transmission process of P-waves across a single uncoupled joint. And the following primary conclusions are thereby arrived at:

(1) The g-λ model specifically for a single uncoupled joint is presented and introduced. Moreover, the finite difference expressions of the particle velocity for P-waves traversing through jointed rock masses are deduced in the context of MC and DDM when the value of λ is not less than 1. Based on the Lemaitre equivalent strain assumption within the realm of rock damage mechanics, the expressions of the stiffness transmission and the stiffness reflection coefficients are deduced.
(2) When a half-sine P-wave is utilized as the incident wave, we noticed that the PPV increases as the parameter λ increases. The shape of the transmitted wave bears a resemblance to that of the incident wave, but with a discernible phase delay. These findings illustrate the practical viability and feasibility of adopting the g-λ model to analyze the nonlinear behavior within the uncoupled jointed rock mass when P-waves propagate through the joint. We can further explore different types of incident waves and their effects on the transmitted and reflected waves, and how these behaviors change with varying parameters and conditions.
(3) The energy transmission shows an increasing trend with higher amplitudes of the incident wave, while it decreases with higher frequencies. The energy transmission also experiences an increase as the value of λ rises. When the frequency remains constant, the stiffness transmission coefficient goes up and the stiffness reflection coefficient goes down as the value of λ increases. If the parameter λ is taken as a larger value, the shape of the transmitted wave will undergo distortion due to the plastic deformation of the rock mass. It can thus be inferred that the parameter λ, which serves as a crucial factor in our model, can potentially be expressed by the plastic work or plastic deformation of the rock joints.

These conclusions may contribute to the prediction of the location of compressed faults ahead of the tunnel face during the excavation of mountain tunnels. In practical engineering, the development status of rock joints is highly complex, and the applicability of the g-λ model to a greater number of rock joints still requires further investigation.

## Supporting information

S1 Appendix (PDF)
