# Supplementary material for: Propagation characteristics of P-wave incident on a single uncoupled joint based on g-λ model
Source: PLoS One. 2024 Dec 5;19(12):e0311359. doi: 10.1371/journal.pone.0311359 (PMC11620657; doi:10.1371/journal.pone.0311359)
Supplement: S1 Appendix — (PDF) [file pone.0311359.s001.pdf]

Appendix A

According to Fig. 4(b), along the lines ab, ac, and cd, the quantities are represented as

$$zv(x_1 + d_n, t) = \sigma(x_1, t) \tag{A1}$$

$$zv(x_1, t) + \sigma(x_1, t) = zp(t - x_1/v_p) + \sigma(0, t - x_1/v_p) \tag{A2}$$

$$zp(t - x_1/v_p) = \sigma(0, t - x_1/v_p) \tag{A3}$$

By adding Eqs.(A2) and (A3) and considering Eq. A1, the following expression can be obtained,

$$v(x_1, t) = 2p(t - x_1/v_p) - v(x_1 + d_n, t) \tag{A4}$$

According to DDM, we have derived the expression for the velocity difference of particles before and after the stress wave passes through the joint as follows,

$$\begin{aligned} v(x_1, t) - v(x_1 + d_n, t) =& \frac{(\frac{\lambda\sigma_n}{d_{ma}k_{ni}} + 1)^{-\frac{\lambda+1}{\lambda}}}{k_{ni}} \frac{\partial \sigma_n}{\partial t} \\ =& \frac{(\frac{\lambda\sigma_n}{d_{ma}k_{ni}} + 1)^{-\frac{\lambda+1}{\lambda}}}{k_{ni}} \frac{z\partial v(x_1 + d_n, t)}{\partial t} \end{aligned} \tag{A5}$$

Substituting Eq. A4 into Eq. A5 yields the following expression,

$$\begin{aligned} \frac{\partial v(x_1 + d_n, t)}{\partial t} =& \frac{2k_{ni}}{z} \left[p(t - x_1/v_p) - v(x_1 + d_n, t) \right] \cdot \\ & \left[\frac{\lambda z v(x_1 + d_n, t)}{d_{ma}k_{ni}} + 1 \right]^{\frac{\lambda+1}{\lambda}} \end{aligned} \tag{A6}$$
